# Activity and Identification of Methanotrophic Bacteria in Arable and No-Tillage Soils from Lublin Region (Poland)

**DOI:** 10.1007/s00248-018-1248-3

**Published:** 2018-09-01

**Authors:** Anna Szafranek-Nakonieczna, Agnieszka Wolińska, Urszula Zielenkiewicz, Agnieszka Kowalczyk, Zofia Stępniewska, Mieczysław Błaszczyk

**Affiliations:** 1 Department of Biochemistry and Environmental Chemistry, Institute of Biotechnology, The John Paul II Catholic University of Lublin, 1 I Konstantynów Str, 20-708 Lublin, Poland; 2 Department of Microbial Biochemistry, Institute of Biochemistry and Biophysics PAS, 5a Pawińskiego Str, 02-106 Warsaw, Poland; 3 Department of Microbial Biology, Warsaw University of Life Sciences, Nowoursynowska 159 Str, 02-776 Warsaw, Poland

**Keywords:** Methane oxidation, Methanotrophs, Arable soils, No-tillage soils, Next-generation sequencing

## Abstract

Methanotrophic bacteria are able to use methane (CH_4_) as a sole carbon and energy source. Photochemical oxidation of methane takes place in the stratosphere, whereas in the troposphere, this process is carried out by methanotrophic bacteria. On the one hand, it is known that the efficiency of biological CH_4_ oxidation is dependent on the mode of land use but, on the other hand, the knowledge of this impact on methanotrophic activity (MTA) is still limited. Thus, the aim of the study was to determine the CH_4_ oxidation ability of methanotrophic bacteria inhabiting selected arable and no-tillage soils from the Lublin region (*Albic Luvisol*, *Brunic Arenosol*, *Haplic Chernozem*, *Calcaric Cambisol*) and to identify bacteria involved in this process. MTA was determined based on incubation of soils in air with addition of methane at the concentrations of 0.002, 0.5, 1, 5, and 10%. The experiment was conducted in a temperature range of 10–30 °C. Methanotrophs in soils were identified by next-generation sequencing (NGS). MTA was confirmed in all investigated soils (in the entire range of the tested methane concentrations and temperatures, except for the arable *Albic Luvisol*)*.* Importantly, the MTA values in the no-tillage soil were nearly two-fold higher than in the cultivated soils. Statistical analysis indicated a significant influence of land use, type of soil, temperature, and especially methane concentration (*p < 0.05*) on MTA. Metagenomic analysis confirmed the presence of methanotrophs from the genus *Methylocystis (Alphaproteobacteria*) in the studied soils (except for the arable *Albic Luvisol*). Our results also proved the ability of methanotrophic bacteria to oxidize methane although they constituted only up to 0.1% of the total bacterial community.

## Introduction

Methane (CH_4_) is the second of the most important greenhouse gases after carbon dioxide (CO_2_). It has 28-fold higher potential than CO_2_ to trap heat radiation on a molecular basis over a 100-year time scale. Its annual emission to the atmosphere (ca. 580 Tg) from industries constantly increases [1, 2]. Its presence in the atmosphere is connected with the metabolism of methanogenic *Archaea* in an anaerobic, organic matter- and nutrient-rich environment and results from human activity. The main role as a natural source of CH_4_ is played by wetlands, termites, and hydrates. Rice plantations, livestock operations, landfills, wastewater treatment, and biomass burning and energy systems are anthropogenic sources. Anthropogenic emissions account for up to 80%, whereas the amount of CH_4_ emitted from natural sources is between 20 and 47% of total emissions [3]. Sabrecov et al. [4] have indicated that a significant part of atmospheric CH_4_ is removed via tropospheric oxidation by hydroxyl radical (OH), which accounts for 85–90% of the estimated annual mean sink (570 Tg CH_4_). The rest of atmospheric CH_4_ can be oxidized by methanotrophic bacteria inhabiting aerated soils. Methanotrophs take up CH_4_ directly from the atmosphere: 1 to 10% is oxidized in this way [4–7]. Methanotrophic activity (MTA) protects against an increase in the CH_4_ concentration in the atmosphere by 50% compared to the initial state [5]. Therefore, the investigation of soil methanotrophic potential and modifying factors is very important when climate changes are discussed and biogeochemical models quantifying consumption of atmospheric CH_4_ are constructed [7–9].

Methanotrophs, i.e., bacteria able to use this gas as a sole carbon and energy source, are natural biological reducers of atmospheric CH_4_. Aerobic methanotrophs are a subgroup of methylotrophic bacteria, which are phylogenetically located in *Alphaproteobacteria*, *Gammaproteobacteria*, and *Verrucomicrobia* [10]. Based on carbon assimilation pathways, phylogeny, chemotaxonomy, and internal membrane structure, methanotrophs have been divided into two types. Both methanotrophic type I and type II typically inhabit aerobic interfaces between anoxic and oxic zones of methanogenic environments such as natural wetlands or rice paddies and reduce the potential of CH_4_ flux to the atmosphere by up to 90% [11]. Type I contains generally low-affinity methanotrophs that are characterized by high methanotrophic capacity (work at a mixing ratio higher than 40 ppm) and are predominant in CH_4_-rich and oxygen-poor environments [12, 13]. In contrast, type II is characterized by low-capacity MTA (able to oxidize CH_4_ at a mixing ratio below 40 ppm); therefore, its representatives are prevalent mostly in CH_4_-poor but oxygen-rich environments. This division suggests that different methanotrophs can be involved in CH_4_ oxidation in wetlands and lowland soils.

Type I contains members of the family *Methylococcaceae* (class *Gammaproteobacteria*), while type II includes representatives of *Methylocystaceae* and *Beijerinckiaceae* from the class *Alphaproteobacteria* [5]. Additionally, type I methanotrophs predominate in a nutrient-rich environment (copiotrophs), whereas type II is present in environments with limited levels of nitrogen [12].

Aerobic methane oxidation is catalyzed by the methane monooxygenase enzyme (MMO), which can be in either particulate (pMMO) or soluble (sMMO) forms, depending on the copper availability in the environment. It has been reported that some methanotrophs can mobilize and acquire copper from mineral and organic solid phases by releasing the fluorescent chromopeptide methanobactin [14, 15].

Importantly, the pMMO form is found in all known methanotrophs except for the genus *Methylocella*, while the sMMO form is not as common and its presence has been confirmed in only a few methanotrophic strains: *Methylococcus*, *Methylosinus*, *Methylocystis*, *Methylomonas*, and *Methylocella* [11, 16–18].

Soil management and different practices, such as agriculture or forestry, can alter the physical and chemical properties of lowland ecosystems, and, in consequence, modify soil microbial activity and methane oxidation potential. Various factors, e.g., soil bulk density, diffusivity, structure, moisture, temperature, and pH are reported as regulatory agents of methane uptake by soils [8, 12, 19]. Bulk density, structure, diffusivity, and moisture determine the possibility of gas exchange between soil and atmosphere, i.e., CH_4_ and O_2_ availability to soil methanotrophs [5]. A suggested by some researchers, the N status, especially that of the NH_4_^+^ form, is connected with CH_4_ oxidation capacity as well, but its function is not clear: according to some data, N can stimulate, inhibit, or exert no influence on soil CH_4_ capacity [20–23].

Methanotrophic activity in soil is extremely important not only in terms of the CH_4_ greenhouse potential but also from the ecological point of view. Methylotrophs, including methanotrophs, are involved in phosphorus acquisition, N-fixation, phytohormone production, iron chelation, and plant growth promotion [24]. Many previous studies have documented the methanotrophic potential of peat soils, rice paddies, and forest soils [10, 19, 25–27]. Recently, the CH_4_ capacity of agricultural soils has focused more attention but, despite the environmental significance of this phenomenon, there have been few attempts to assess the impact of tillage on CH_4_ consumption by lowland mineral soils. Little is still known about how soil uses modified MTA because of lack of comparable measurements in pedogenetically related soils representing the same type as agricultural soils. Moreover, forest soils are often used as control soils in respect to arable sites. However, forest soils are characterized by a different plant and microbial composition than agricultural ones; therefore, they are not a suitable background [28]. Furthermore, there are very few data on identification of methanotrophic bacteria in both agricultural and uncultivated soils, which is required for complete characteristics of CH_4_ oxidation phenomena. Importantly, methanotrophy in mineral soils may differ from that in organic soils due to the different substrate availability, soil structure, and watering condition. Additionally, it may vary in soils originating from particular regions due to the unquestionable effect of climatic conditions. Therefore, the aim of our study is to find the answers to the following questions: (1) How does MTA change across types of soil representative for Poland? (2) How does the land use mode modify MTA? (3) What is the response of methanotrophic bacteria to an increase in the CH_4_ concentration in the atmosphere? (4) Is this response similar in all soil types? (5) Which methanotrophs are involved in CH_4_ oxidation in Polish mineral soils?

## Methods

### Experimental Sites and Soil Sampling

Four samples of agricultural soils (code A) and four no-tillage (control) soils (code NT) were taken from the surface layer (0–20 cm) in April 2014 from Lubelskie district (51° 13′ N 22° 54 ′E). The studied area was described in our previous studies [29, 30]. The sampling points were chosen on the basis of soil identification performed in 1991 during the creation of the Bank of Soil Samples (BSS) by researchers from the Institute of Agrophysics, Polish Academy of Science in Lublin [30, 31]. The very precise description of the sampling points cataloged in the BSS database (name of place and geographic coordinates) facilitated a precise return to the sampling sites [29].

10 × 10-m squares were chosen (according BSS database) from each of the four selected sampling points. A minimum of 50 random soil samples were taken from each location using a 2.5-cm-diameter auger, strictly complying with the rules included in Polish Norm for soil sampling [32, 29]. Single samples were combined and homogenized into one sample in order to receive the most representative soil material for each investigated site [29]. In this way, we obtained four samples from the arable (A) soils and 4 from the no-tillage (NT) soils. Importantly, control samples (non-agriculturally used and non-forested sites covering at least a 1-ha area) belonged to the same soil type as arable soils and were located in the nearest neighborhood to the arable soil sampling sites. The investigated soil material was classified according to FAO as *Albic Luvisol*, *Brunic Arenosol*, *Haplic Chernozem*, and *Calcaric Cambisol* (Table 1). In laboratory conditions, each sample was passed through a 2.0-mm sieve to remove large pieces of rocks and plant material and shortly stored at 4 °C prior to analysis [34].

**Table 1:** Location of the sampling sites in the Lublin region

Soil no.	Soil type (FAO)	Soil orders (WRB)*	Geographic coordinates	Village	Crop
1	*Albic Luvisol*	*Luvisols*	22° 10′ 17.7″ 51° 26′ 24.6″	Dęba	Oat
2	*Brunic Arenosol*	*Arenosols*	22° 15′ 55.5″ 51° 23′ 1.9″	Markuszów Osada	Oat
3	*Haplic Chernozem*	*Chernozems*	23° 42′ 56.6″ 50° 44′ 48.3″	Hostynne	Triticale
4	*Calcaric Cambisol*	*Cambisols*	23° 11′ 43.9″ 51° 12′ 10.8″	Brzeziny	Oat

*IUSS World Reference Base for Soil Resources [33]

### Soil Characteristics

The soil reaction (pH), redox potential (Eh), and electric conductivity (EC) were determined in a soil suspension in distilled water (1:2, in triplicate) using a multifunctional potential meter and electrodes: a glass electrode (Cartrode pH E16M340), a combined platinum and an Ag/AgCl (E31M004) electrode, and an EC conductivity cell (CDC 30T-3) (Radiometer Analytical S.A., France). Carbon forms were determined by means of TOC-VCSH with an SSM-5000A module (Shimadzu, Japan). The amount of total organic carbon (TOC) was calculated from the difference of total carbon (TC) and inorganic carbon (IC) [35, 36].Soil moisture was determined with the gravimetric method (24 h, 105 °C) [37].Concentrations of biogenic nitrogen compounds were determined using AutoAnalyzer3 (Bran + Luebbe, Germany) in soil extraction (35 g fresh soil and 100 ml deionized water) [38].

### Methane Oxidation and Methanotrophic Activity

Ten grams of soils were placed in 60-ml serum vials and closed with butyl rubber septa and caps. Triplicates of each soil sample were incubated at CH_4_ concentrations of 0.002, 0.5, 1.0, 5.0, and 10.0% (*v*/*v*) and temperatures of 10, 20, and 30 °C, up to 90 days. Consumption of CH_4_ and O_2_ in the headspace was determined with the gas chromatography technique (GC 3800, Varian, USA) equipped with flame ionization (FID, 200 °C) and thermal conductivity (TCD, 120 °C) detectors in series and with the use of two types of columns: PoraPlot Q 0.53 mm ID (25 m) and a molecular sieve 5A 0.53 mm ID (30 m) connected together. Helium was used as the carrier gas [27, 35]. The detector responses were calibrated using series of gas standards: CH_4_ (Linde, Poland), and O_2_ (Air Products, Poland) in helium and nitrogen, respectively. The MTA rate was determined on the basis of CH_4_ reduction in time and calculated as the dry mass of the investigated soils and time (μMol CH_4_/kg d.w./day). Statistical processing of data (ANOVA, Tukey’s test) was performed using Statistica software [27, 39].

The Q10 temperature coefficient values used to compare the rates of biological reactions or processes were calculated for 10–20 °C and 20–30 °C increments; their average values are presented [40].

### DNA Extraction and NGS Procedure

DNA extraction was carried out no later than 24 h after sample collection according to the protocol described by Tomczyk-Żak et al. [41]. A modification step involving an additional purification stage by CsCl gradient centrifugation (16 h, 70,000 rpm, 20 °C; Sorvall WX Ultra Thermo Scientific) was added. The whole DNA extraction procedure was described in detail in Wolińska et al. [37].

Next-generation sequencing (NGS) with the Ion Torrent™ technology (Ion PGM™, Life Technologies) was used for metagenomic analysis. The Ion Plus Fragment Library Kit, RT-PCR Ion Universal Library Quantitation Kit, Ion PGM™ Template OT2 400 Kit, and Qubit™ Fluorometric Quantitation Kit were applied for metagenomic 16S rRNA amplicons. The sequencing step (Ion 318™ Chip Kit v2) was carried out in the Laboratory of Microarrays Analyses (IBB PAS, Warsaw) according to manufacturer’s instructions [40].

Bioinformatic analysis was performed using MOTHUR v.1.34.4. [42]. Raw reads were extracted from the fastq files to filter out the V3 region. All the reads were dereplicated and aligned to the mothur-formatted version (silva.nr_v119), as recommended by Quast et al. [43]. Chimeras were removed using UCHIME implementation [44]. After that, the sequences were clustered into operational taxonomic units (OTUs) based on a 99% similarity threshold [30].

## Results

### Chemical Properties of Soils

The pH value in the studied soils ranged from 4.78 to 7.22 and the most acidic conditions were found in *Brunic Arenosols* (Table 2). In general, arable soils (A) were by 0.18–1.04 units more acidic than no-tillage soils (NT). Both A and NT soils were well aerated and their redox potential (Eh) ranged from 435.2 to 561.3 mV. The highest moisture was found in *Haplic Chernozem* (31.03%), and this factor generally achieved higher levels in the no-tillage soils. Furthermore, the NT soils were richer in the TOC content (by approx. 0.47%) than the A soils. In contrast, soils A were characterized by even 4-fold higher salinity, as in the case of *Calcaric Cambisol*, in relation to the NT sites. The ammonia nitrogen (N-NH_4_) concentration was relatively low; it reached 3.39 mg/kg only in the case of no-tillage *Calcaric Cambisol*, while N-NO_3_ was the dominant form in the other NT soils. In turn, both ammonia and nitrate concentrations were generally higher in the NT than A soils, with the exception of *Albic Luvisol* (Table 2).

**Table 2:** Chemical properties of no-tillage (NT) and arable soils (A): 1-*Albic Luvisol*, 2-*Brunic Arenosol*, 3-*Haplic Chernozem*, and 4-*Calcaric Cambisol* from Lublin region (± SD)

Soil no.		pH (H_2_O)	EC (mS/cm)	Moisture (%)	Eh (mV)	TOC (%)	N-NH_4_ (mg/kg)	N-NO_3_ (mg/kg)	N-NO_2_ (mg/kg)
1	NT	6.27 ± 0.01	0.034 ± 0.003	9.76 ± 0.11	435.2 ± 0.2	1.76 ± 0.12	0.09 ± 0.006	1.68 ± 0.014	0.17 ± 0.001
	A	5.23 ± 0.06	0.045 ± 0.08	8.20 ± 0.20	477.4 ± 0.4	0.98 ± 0.003	0.01 ± 0.006	9.34 ± 0.8	0.11 ± 0.003
2	NT	5.58 ± 0.04	0.049 ± 0.001	8.63 ± 0.15	480.2 ± 17.75	2.06 ± 0.20	0.69 ± 0.009	10.18 ± 0.14	0.21 ± 0.002
	A	4.78 ± 0.006	0.063 ± 0.09	9.23 ± 0.06	480.6 ± 0.17	0.83 ± 0.095	0.01 ± 0.007	20.26 ± 0.07	0.09 ± 0.004
3	NT	7.22 ± 0.02	0.059 ± 0.006	31.03 ± 0.23	529.2 ± 0.23	4.88 ± 0.145	0.02 ± 0.002	8.23 ± 0.02	0.44 ± 0.006
	A	6.61 ± 0.05	0.123 ± 0.05	24.66 ± 0.28	561.3 ± 0.36	1.64 ± 0.026	0.02 ± 0.001	27.43 ± 0.08	0.09 ± 0.003
4	NT	5.76 ± 0.01	0.040 ± 0.002	12.50 ± 0.17	493.8 ± 0.2	1.59 ± 0.125	3.39 ± 0.06	10.12 ± 0.07	0.09 ± 0.004
	A	5.58 ± 0.06	0.168 ± 0.05	10.86 ± 0.11	503.9 ± 0.2	0.97 ± 0.064	0.05 ± 0.01	77.17 ± 0.14	0.08 ± 0.007

### Methanotrophic Activity

**Fig. 1:**
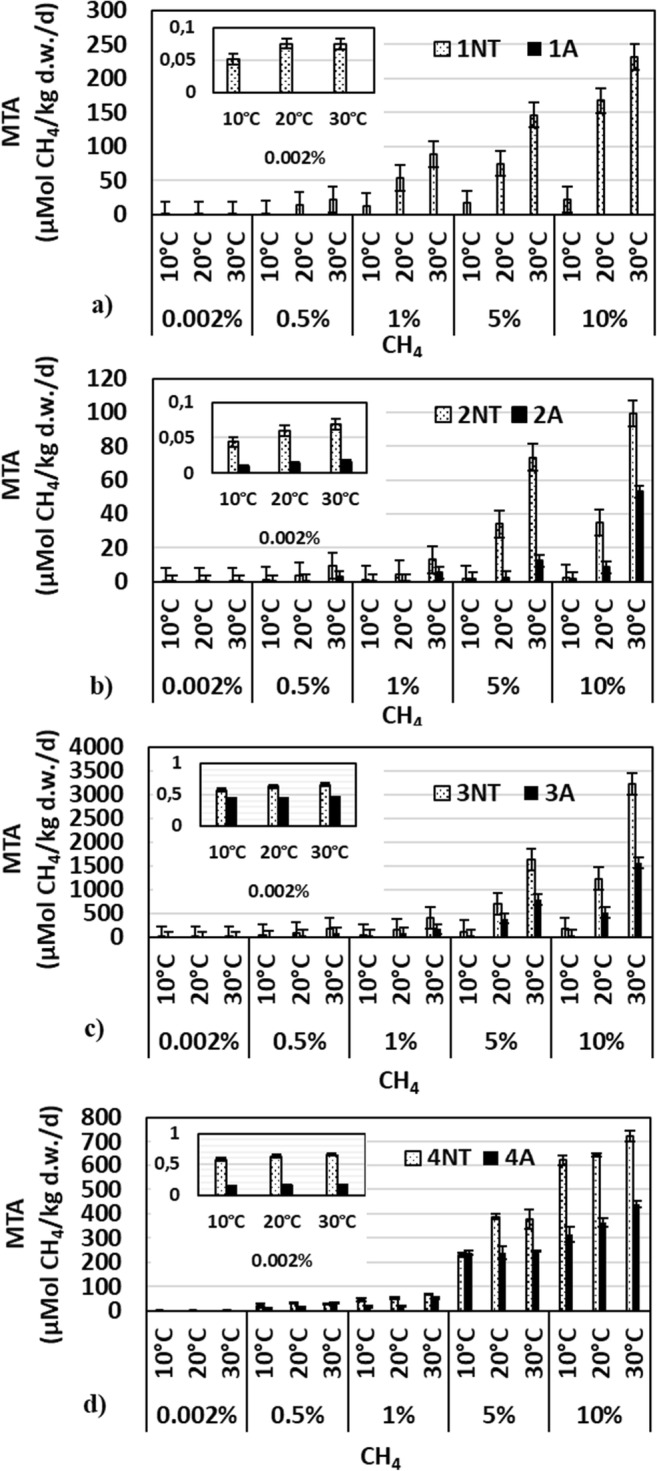
Methanotrophic activity in no-tillage (NT) and agricultural (A) soils: 1-*Albic Luvisol* (**a**), 2-*Brunic Arenosol* (**b**), 3-*Haplic Chernozem* (**c**), and 4-*Calcaric Cambisol* (**d**), incubated at different CH_4_ concentrations (0.002%; 0.5%; 1%, 5%, and 10% *v*/*v*), at different temperatures (10, 20, and 30 °C). Mean values with standard error (SE) are presented

Methane oxidation was observed in all investigated soils, with the exception of the A *Albic Luvisol* (Fig. 1a), both in the wide range of substrate availabilities (from 0.002 to 10% CH_4_*v*/*v*) and a temperature range of 10–30 °C (Fig. 1). MTA varied from 0.05 to ca. 3200 μMol CH_4_/kg d.w./day depending on the soil type and the experimental conditions (Fig. 1).

*Haplic Chernozem* was characterized by the highest MTA levels, even above 3200 and 1550 μMol CH_4_/kg d.w./day in the A and NT soils, respectively (Fig. 1c). In turn, the lowest methane capacity was found in *Brunic Arenosol*, where it amounted maximally to 99 and 53 μMol CH_4_/kg d.w./day (Fig. 1b).

MTA in *Calcaric Cambisol* reached 710 μMol CH_4_/kg d.w./day. In this soil, MTA did not significantly differ between the temperature combinations at the initial CH_4_ concentration (Fig. 1d). The statistical analysis of the data indicated that MTA was significantly affected by soil tillage, substrate availability, soil type, and temperature (Table 3). These differences were found both in data collected for single soils and in the entire data set. Moreover, temperature sensitivity expressed as a Q10 coefficient indicates a stronger effect of the temperature range increasing from 10 to 20 °C than from 20 to 30 °C, when the maximal Q10 factors oscillated between 9.7 and 8.6, respectively (Table 4). MTA in optimal conditions (30 °C and 10% CH_4_*v*/*v*) was strongly stimulated by the increase in pH, Eh, TOC, moisture, and N-NO_2_ (Table 5).

**Table 3:** Influence of sample properties on methane oxidation capacity in no-tillage (NT) and arable soils (A): 1*-Albic Luvisol*, 2*-Brunic Arenosol*, 3*-Haplic Chernozem*, and 4*-Calcaric Cambisol*

Range of analyzed data	Parameters			
	Temperature (°C)	Initial concentration of CH_4_ (%)	Way of use	Soil type
All data	***	***	**	***
Arable soils	***	***		***
No-tillage soils	***	***		***
Soil no. 1	*	***	***	
Soil no. 2	***	***	***	
Soil no. 3	***	***	**	
Soil no. 4	*	***	***	
Soil no. 1NT	***	***		
Soil no. 1A	na	na		
Soil no. 2NT	*	*		
Soil no. 2A	***	***		
Soil no. 3NT	***	***		
Soil no. 3A	***	***		
Soil no. 4NT	***	***		
Soil no. 4A	***	***		

Tukey’s analysis; *p < 0.05; **p < 0.01; ***p < 0.001; na—no active

**Table 4:** Temperature sensitivity of MTA expressed as Q10 values in no-tillage (NT) and arable soils (A): 1-*Albic Luvisol*, 2-*Brunic Arenosol*, 3-*Haplic Chernozem*, and 4-*Calcaric Cambisol*

Sample code		Q10 ^(20–10°C)^					Q10 ^(30–20°C)^				
		Initial methane concentration (%)									
		0.002	0.5	1	5	10	0.002	0.5	1	5	10
1	NT	1.47	5.15	4.27	4.47	7.46	1.1	1.56	1.66	1.95	1.38
	A	–	–	–	–	–	–	–	–	–	–
2	NT	1.35	2.17	1.28	1.35	4.82	1.16	4.31	8.66	4.96	6.30
	A	1.48	3.98	3.28	9.73	9.32	1.18	2.61	2.82	2.16	2.84
3	NT	1.1	2.39	3.27	5.83	7.28	1.05	2.25	2.52	2.31	2.60
	A	1.14	2.19	2.33	9.67	9.65	1.02	2.03	1.96	2.03	2.99
4	NT	1.11	1.53	1.12	1.68	1.04	1.05	1.0	1.29	0.97	1.12
	A	1.01	1.34	1.20	1.0	1.16	1.06	2.61	2.80	1.03	1.21
Average NT		1.29	2.81	2.49	3.33	5.15	1.07	1.07	2.26	3.53	2.55
Average A		1.17	2.51	2.27	6.80	6.71	1.08	1.08	2.41	2.53	1.74

**Table 5:** Correlations between the investigated soil properties and MTA in no-tillage (NT) and arable soils (A) (*R* coefficient, *n* = 24)

Range of data	pH	Eh	TOC	Moisture	EC	N-NH_4_	N-NO_3_	N-NO_2_
NT	0.837***	0.859***	0.947***	0.996***	0.543 ns	− 0.493 ns	0.200 ns	0.858***
A	0.957***	0.998***	0.989***	0.989***	0.534 ns	− 0.068 ns	0.126 ns	0.201 ns
All	0.832***	0.709***	0.947***	0.974***	0.265 ns	− 0.079 ns	0.111 ns	0.740***

Regression analysis: * *p* < 0.05; **p < 0.01; ***p < 0.001; ns—not significant

### Identification of Methanotrophic Bacteria

**Fig. 2:**
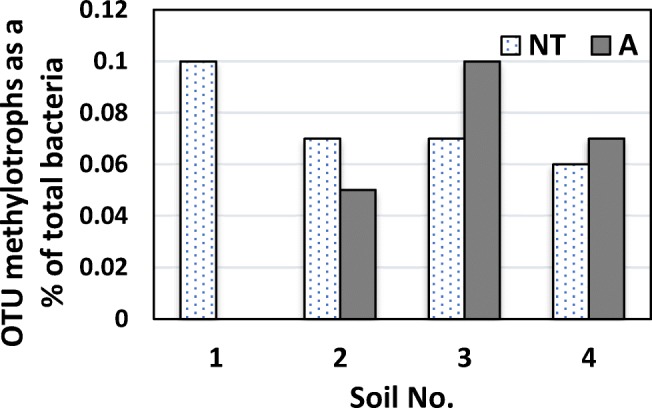
Methanotroph OTU expressed as % of the total bacteria detected in the investigated no-tillage (NT) and agricultural (A) soils: 1-*Albic Luvisol*, 2-*Brunic Arenosol*, 3-*Haplic Chernozem*, and 4-*Calcaric Cambisol*

Only up to 1 OTU of methanotrophs was found in the investigated soil samples. Considering the total microbial community, methane-oxidizing bacteria accounted maximally for 0.1% of soil bacteria (Fig. 2). The proportion of methanotrophs among all detected bacteria was greater in the A soils in the case of *Haplic Chernozem* and *Calcaric Cambisol* (Fig. 2) and in NT soils in *Brunic Arenosol*. In the case of *Albic Luvisol*, methanotrophs were identified only in soil that was not used for agricultural purposes. The metagenomic analysis showed that the methanotrophic bacteria in all studied soils were classified as members of the genus *Methylocystis*.

## Discussion

Next-generation sequencing (NGS) of environmental samples is an important technique applied in ecology or soil science. It offers a possibility to examine the structure and diversity of microbial communities directly from samples without the necessity of bacterial culture in laboratory conditions. This makes NGS very attractive for investigation of the diversity of microbial species and provides understanding of microbial connections in a broader and deeper perspective. To date, methanotrophs in soils have most frequently been detected by sequencing products of PCR reaction with application of specific gene primers (*pmoA*) or 16S rRNA, FISH, and DGGE of DNA isolated directly from soils or from cultures (Table 6). Currently, the application of NGS based on the use of universal primers is a guarantee of receiving a huge quantity of data and thus seems to be an adequate way for identification of soil methanotrophs in their environment; this approach was applied in this study.

**Table 6:** Examples of various environments in which *Methylocystis* were detected among other methanotrophs

Region	Environments/soil type	Methanotrophs	pH	Methods of identification	Literature
Tierra del Fuego, Argentina	Sphagnum bog	*Methylocystis*, *Methylosinus Methylomonas*	3.5–4.6	*pmoA* coloning, sequencing	[45]
Minnesota	Boreal oligotrophic peatland	*Methylocystis*, *Methylomonas*, *Methylovulum*	3.5–4.0	*pmoA* targeted NGS	[2]
Poland, Lublin region	High moor peat	*Methylocystis Methylosinus*	4.43	Sequencing of 16S rRNA fragments specific for methanotrophs	[46]
China, Jiangsu	Rice paddy field	*Methylococcus Methylocaldum* (both type I), and *Methylocystis* (type II),	Not given	*pmoA*, T-RFLP^a^ analysis, cloning, sequencing	[47]
Germany	Forest, meadow, pasture	*Methylocaldum* *Methylosinus* *Methylocystis*	4.6–8.0	DGGE^b^*pmoA* bands sequencing, PLFA analysis	[25]
European part of Russia	Unmanaged and managed soils	*Methylosinus* *Methylocystis Methylocaldum Methylobacter*	4.2–8.2	*pmoA* coloning, sequenceing	[19]
Nakorn Ratchasrima province, Thailand	Upland different use soils (forest, reforested, cornfields	*Methylocystis Methylobacter* *Cluster 5*	4.2–5.6	DGGE^b^ pmoA and mmoX bands reamplified and sequencing	[26]
Russia, Moscow	Forest and agricultural soddy-podzolic soils	*Methylocaldum Methylocystis*	4.6–5.3	DGGE^b^*pmoA* bands cloning and sequencing	[10]
Poland, Jastrzębie-Moszczenica, and Bogdanka coal mines	Coal mine	*Methylobacter* *Methylocaldum* *Methylosinus* *Methylocystis*	7.56–8.9	*pmoA* cultured methanotrops, sequencing, FISH^c^	[48, 49]
Poland, Lublin region	No-tillage and agricultural	*Methylocystis*	4.78–7.22	bacterial 16S rRNA gene V3-V4 variable region, NGS	This study

^a^T-RFLP—terminal restriction fragment length polymorphism analysis

^b^DGGE—denaturing gradient gel electrophoresis

^c^FISH—fluorescent in situ hybridization

With the use of NGS sequencing, it was possible to detect methanotrophic bacteria in the investigated soils. The methanotroph abundance was rather low (1 OTU), up to 0.1% (Fig. 2), while maximally 0.37% of total bacterial community has been found in ombrotrophic peat soils [2]. Our earlier studies demonstrated that the total microbial structure in soils from the Lublin region was generally dominated by *Proteobacteria* (57%) and *Bacteroidetes* (20%). The subdominants were represented by *Actinobacteria (*7%), *Firmicutes* (6%), *Acidobacteria* (5%), and *Elusimicrobia* (3%). Less frequently, abundant *Verrucomicrobia* (0.7%), *Planctomycetes* (0.5%), *Cyanobacteria* (0.5%), and *Chlorobi* (0.3%) were noted [29, 30]. All methanotrophs identified in the present study represented the genus *Methylocystis*, belonging to the family *Methylocystaceae* of the phylum *Proteobacteria*, and were classified as type II methanotrophs. The representatives of this genus are aerobic bacteria but some species have been isolated also from microaerophilic environments [50]. Some methanotrophic species among *Methylocystis* are reported to be facultative methanotrophs capable of conserving energy for growth on multicarbon organic acids (e.g., acetate, pyruvate, succinate, malate) and ethanol [51].

Generally, methanotrophs are divided into two groups: high affinity—preferring low concentrations of CH_4_ and low affinity—able to use methane in a high mixing ratio. Complete genome sequences of the bacterial representatives of *Methylocystis* (strain SC2) demonstrated that these species expressed two types of particular monooxygenase (pMMO). The pMMO1 form promotes bacterial growth at a high methane concentration, while pMMO2 in low-methane environments (< 600 ppm) [14, 52–54]. This unique property, i.e., the ability to oxidize methane in a wide range of concentrations, makes *Methylocystis* environmentally well distributed and ecologically most relevant methanotrophic bacteria [53].

The fact that *Methylocystis* species have greater metabolic flexibility than other methanotrophs makes them more ubiquitous [53]. They were found in mineral and organic soils, both cultivated and no-tillage, or peatland and forest soils (Table 6). Therefore, they are able to inhabit environments with different methane concentrations, carbon content, and moisture. Their presence in soil is very valuable from the environmental and ecological point of view, especially in soils that are poor in carbon. Methanotrophs are able to provide other microbes with different carbon compounds, e.g., methanol, proteins (amino acids), polysaccharides, and nucleic acids as methane metabolites, and a methane-driven food web created in this way [51]. Methane-oxidizing bacteria can therefore support the richness of microorganisms inhabiting soils, which is one of the indicators of soil quality and soil conditions. This is an important function especially in agricultural soil that is often characterized by lower carbon content than that in no-tillage soils, as presented in our study (Table 2). Depending of the mode of land use, cultivated soils are more exposed to pollution, which is a source of various chemical components introduced to soils as fertilizers or pesticides [9, 39]. Modern agricultural practices are focused on achievement of the largest possible yield, contributing to the high level of soil pollution. Both no-tillage and agricultural soils are exposed to pollution generated by industry or wastewater as well [55]. Therefore, the presence of methanotrophic bacteria in soil is crucial, as methanotrophs are able to degrade diverse types of heavy metals as well as organic pollutants with their MMO enzymes. It has been shown that MMO can transform a variety of hydrocarbons (alkanes, alkenes) and oxidize a wide range of substances, including aliphatic hydrocarbons, alicyclic hydrocarbons, aromatic compounds (halogenated benzenes, toluene, styrene), and halogenated aliphatic compounds (chloroform, dichloroethene, trichloroethylene, tetrachloroethene, hydrochlorofluorocarbons) [18, 56].

It was clearly evidenced that despite the presence of bacterial representatives of one genus of methanotrophs—*Methylocystis*—in the investigated soil, the activity of these bacteria differed significantly among the soil types. The highest potential for CH_4_ oxidation was found in *Haplic Chernozem*. This type of soil was characterized by the highest pH value, moisture, Eh, and TOC content. The statistical analysis revealed a strong effect of these factors on the methane oxidation potential (Table 5).

The reaction of the tested soils was rather suitable for MTA (4.78–7.22) although it should be mentioned that the requirements of *Methylocystis* for optimal growth include pH approaching to neutral; however, they are also able to grow in a wide range of pH, from 4.5 to 9.0 [57, 58]. Nevertheless, no methane-oxidizing bacteria were detected by NGS and there was no MTA in the agricultural *Albic Luvisol* (pH 5.23). Data obtained from other environments, not only from peat soils which are known for their low reaction but also soils with different land use systems (managed and unmanaged) like meadows, forested, or deforested sites, confirmed both CH_4_ oxidation and the presence of *Methylocystis* in these environments (Table 6).

The function of N fertilizers in soils is still not clear, as they can either stimulate or inhibit CH_4_ oxidation in soils [9]. The concentration of N-NH_4_ in a majority of the studied samples was low and it was significantly higher only in soils NT2 and NT4, i.e., 0.69 and 3.39 mg/kg, respectively (Table 2). Ammonia and aerobic methane-oxidizing bacteria compete for O_2_, which serves as an electron acceptor. The presence of ammonia can inhibit CH_4_ oxidation because the MMO enzyme involved in the first step of CH_4_ oxidation is evolutionarily related to ammonia monooxygenase and able to bind to NH_4_^+^ and react with it, since NH_4_^+^ and CH_4_ are similar in their size and structure [59, 60]. Therefore, the presence of NH_4_^+^ in soils is regarded as an inhibitor of CH_4_ oxidation [61]. However, the involvement of methanotrophs in ammonia oxidation can cause reduction of the availability of ammonia to ammonia oxidizers. The higher level of ammonia estimated in two of the investigated no-tillage soil samples (NT2 and NT4) did not reduce the MTA level in comparison to the agricultural soils, which exhibited significantly lower N-NH_4_ concentrations, probably due to the low concentration of ammonia. The inhibition effect was observed when the N-NH_4_ content exceeded 98 mg/kg, usually immediately after fertilization [62]. A high level of N-NO_3_ (in comparison to N-NH_4_), which is a product of ammonia oxidation, can be a consequence of both the efficient activity of ammonia oxidizers and the Eh level above 400 mV favoring the dominance of this form of nitrogen in the soil environment. In contrast, the low NH^+^_4_ availability in soil, especially arable and generally enriched with N fertilizers, reflected a nitrogen pool that remained in the soil after the previous vegetation season and before the next fertilization cycle, because the soils were sampled in early spring (Table 2). In the A soils, a significantly higher (5.5–7.6-fold) concentration of N-NO_3_ was noted but it did not influence MTA (Table 3).

The positive impact on CH_4_ oxidation was confirmed in the case of Eh and moisture (Table 5). Slightly higher oxidoreduction potential and lower moisture were found in the cultivated soils (Table 2). Soil cultivation can increase soil aeration, which is reflected by higher redox potential values, and concurrently results in a decrease in the soil water content; hence, lower moisture was determined in the agricultural soils (Table 2). The determined values of Eh were above + 300 mV, and soils characterized by such conditions are regarded as well aerated [36]. It should be underlined that the soil samples tested in this study were collected in spring, i.e., in a season of rather high precipitation in Poland. The water retention capability is dependent on soil structure and genesis. Among the investigated soils, the highest moisture was found in *Haplic Chernozem*. In this soil, the main factor of soil formation is grassy vegetation, which provides a supply of organic matter and TOC. Organic matter increases water-holding capacity [63]. Moisture was mostly higher in the NT soils, which is connected with plant debris remaining after the previous vegetation season. Data processing indicated that soil moisture has a high influence on MTA (Tables 2 and 3). The moisture range was estimated between 8.2 to 31%; however, there was no CH_4_ uptake at 8.2% in *Albic Luvisol*. In a landfill cover soil, CH_4_ oxidation has been observed at a moisture level between 8 and 44%, but optimum conditions have been determined to be 20–25% [64, 65]. Since a decrease in the water content increases gas diffusivity and causes osmotic stress, a certain moisture level must be maintained to support microbiological activity.

The activity of bacteria involved in methane oxidation in the four soil orders dominating in Poland, i.e., *Luvisols*, *Arenosols*, *Chernozems*, and *Cambisols*, was strongly enhanced by temperature (Tables 3 and 4, Fig. 1). Generally, the higher the initial CH_4_ concentration is, the stronger the influence of the temperature on soil MTA can be observed. The Q10 values for CH_4_ oxidation are expected to rise when enzyme systems are saturated with the substrate and to decline at limitation of the substrate supply [66]. The lowest effectiveness of temperature and Q10 slightly higher than 1 were detected when CH_4_ was available at the concentration of 0.002% (Table 4). The average Q10 values ranged between 1.07 and 5.15 for the NT soils and between 1.08 and 6.8 for the A soils. The maximal Q10 values, up to 9.7 and 8.66, were calculated for oxidation over the temperatures range of 10–20 °C and 20–30 °C, respectively (Table 4). The increase in the temperature from 10 to 20 °C had a stronger effect on MTA than the increase from 20 to 30 °C; however, the most efficient potential of methane oxidation was detected at 30 °C (Fig. 1). This is in agreement with the results of identification of the methanotrophs, as bacteria from the genus *Methylocystis* detected in the investigated soils are able to grow in a wide range of temperature, from 5 to 40 °C, but their optimal growth is observed at approx. 25–30 °C [58]. The highest thermal sensitivity of methane-oxidizing bacteria in landfill cover soils has been reported at Q10 values in the range of 3.4–4.1 [64, 67] or 6.5–8.4 [65], but little is known about the temperature effectiveness in no-tillage and agricultural soils.

At the initial CH_4_ concentration 0.002% (20 ppm) in the arable soils, MTA achieved values between 0 and 0.5 μMol CH_4_/kg d.w./day. This result was comparable to that obtained for arable soils classified as soddy-podzolic soils (*Podzoluvisols*, *Haplic Phaeozem)* (according to the FAO classification), where the activity was estimated at up to 0.025 and between 0.125 and 0.55 μMol CH_4_/kg d.w./day [10, 68].

The agricultural and no-tillage soils displayed differences in their chemical properties. Lower pH and TOC content but higher salinity (EC) and N-NO_3_ concentration were exhibited by the arable soils in comparison to the no-tillage soils. This divergence is commonly known as one of the symptoms of the soil degradation process. The changes in soil chemistry and biology mentioned above are a consequence of different agricultural practices. Based on the current study, it is evident that soil MTA in the selected Polish soils is strongly modified by their agricultural use both in the case of this particular type of soils and in all soils available in the database (Table 3, Fig. 1). In the NT soils, MTA was on average 1.8 to 4.4-fold higher than in the A soils. At room temperature (20 °C), MTA was 7-fold higher in *Brunic Arenosols*, and approx. 2-fold higher in *Haplic Chernozem* and *Calcaric Cambisol* in the NT soils than in the A soils (Fig. 1). In comparison to soils from the European part of Russia, which are genetically classified as *Luvisols*, *Podzoluvisol*, and *Chernozems*, MTA was from 1.6 to 4.4-fold higher in natural than agricultural soils [19]. Interestingly, the Russian agricultural *Luvisols* were metanotrophicaly active while no methane oxidation capacity was detected in the *Luvisols* analyzed in the present study. Additionally, this fact was supported by the NGS data, according to which no methanotrophs were identified in *Albic Luvisol*. In turn, among four agricultural *Luvisols* (deciduous forest, pasture, and two soils with wintergrain cultivation) studied in Germany by Knief et al. [25], methane oxidation (from atmospheric to 400 ppm CH_4_ concentration) was observed in three soils (one of the farmland soils was not active), despite the fact that methanotrophs were detected in all of these soils. Representatives of *Methylosinus* dominated. In forest *Cambisols*, MTA was 7.8-fold higher than in agricultural *Luvisols*. In the present study, *Calcaric Cambisol* was metanotrophically active and the MTA was almost 3-fold higher in NT *Calcaric Cambisol* than in *Albic Luvisol* (Fig. 1).

The influence of soil cultivation on methane oxidation capacity is very important for controlling soils. The use of no-tillage soils as controls of agricultural soils leads to formulation of an erroneous conclusion that agricultural soils represent higher potential for methane uptake. For example, the MTA in *Brunic Arenosol* reached maximally 100 μMol CH_4_/kg d.w./day, which is significantly lower than this value for arable *Calcaric Cambisol* or *Haplic Charnozem* (Fig. 1).

Afforested sites are often used as control soils [19, 25, 26]. Irrespective of its stage of development, afforestation leads to an increase in the methanotrophic potential, as shown in the case of sandy loam, with a significant impact of carbon and moisture on MTA [28]. The control in the present study comprised soil sites that were not forested and not arable, but represented the same type as the agricultural soils. We demonstrated in all cases that the arable soils always represented a decreasing methane uptake. The lower CH_4_ uptake noted in the arable soils was connected with an increase in CH_4_ emission. This dangerous impact might lead to intensification of the greenhouse effect because the methane concentration in atmosphere is constantly growing [69].

## Conclusions

Among the types of soils investigated in this study, the highest methane oxidation potential was found in *Haplic Chernozem*. Our results demonstrated a negative effect of soil cultivation on the CH_4_ oxidation potential in different types of soils. In the arable soils, the ability to uptake methane was 2-fold lower or eliminated (as shown in the case of *Albic Luvisol*). The increase in the initial CH_4_ concentration in all tested soils significantly modified MTA and resulted in its rise. Moreover, we have shown that soil methanotrophic bacteria are able to oxidize methane in a wide range of its concentration (0.002–10% *v*/*v*), which is very important in the context of climate change. The NGS technique allowed identification of methanotrophs from the genus *Methylocystis*, which accounted for approx. 0.1% of the total soil bacterial community.

In investigations of the influence of agricultural soil use on methanotrophic activity, it is very important to choose proper no-tillage soils that can serve as controls. To achieve reliable results, comparison of soils representing the same type in accordance with the genetic classification and with a similar type of vegetation seems to be important. To our knowledge, this is the first report giving evidence of the impact of agriculture on the methanotrophic potential of soils distinguished on the basis of the FAO classification and confirming the identification of methanotrophic bacteria involved in methane oxidation. Last but not least, the effect of soil chemical factors on methanotrophic activity in agricultural and control soils was determined. The significant impact of temperature, type of land use, type of soils, pH, Eh, moisture, and TOC on MTA was statistically evidenced.

Based on the presented results and given the global importance of the ability of arable and no-tillage soils to remove CH_4_ from the air, investigations of other types of soils and the response of methanotrophs to the observed temporary drought are needed.
